# Predicting Ultra-High Risk Outcomes Using Linguistic and Acoustic Measures From High-Risk Social Challenge Recordings: mHealth Longitudinal Cohort Exploratory Study

**DOI:** 10.2196/75960

**Published:** 2025-12-30

**Authors:** Samuel Ming Xuan Tan, May Yen Lieu, Jun Kai, Zixu Yang, Luke KK, May O Lwin, Jimmy Lee, Wilson Wen Bin Goh

**Affiliations:** 1 LKC School of Medicine, Nanyang Technological University, 59 Nanyang Drive, Experimental Medicine Building, Singapore, 636921, Singapore, 65 65927871; 2 School of Humanities, Nanyang Technological University, Singapore, Singapore; 3 Institute of Mental Health, Singapore, Singapore; 4 WKW School of Communications, Nanyang Technological University, Singapore, Singapore

**Keywords:** machine learning, mental health, outcome prediction, psychosis, speech data, ultra-high risk

## Abstract

**Background:**

Early detection of individuals at ultra-high risk (UHR) for psychosis is critical for timely intervention and improving clinical outcomes. However, current UHR assessments, which rely heavily on psychometric tools, often suffer from low specificity. Speech-based machine learning prediction models can potentially be used to improve prognostic accuracy. However, existing studies often used long, open-ended speech tasks, which limit scalability. The High-Risk Social Challenge (HiSoC) is a short 45-second speech task designed to measure social functioning in individuals with UHR. If the HiSoC task is able to capture predictive signals, it may serve as an effective and scalable speech task for future prediction models.

**Objective:**

The study aims to explore whether linguistic and acoustic features extracted from the HiSoC task are associated with UHR outcomes and if they are predictive of different UHR outcomes.

**Methods:**

Audio recordings of HiSoC task responses were collected from 41 participants with UHR enrolled in the Longitudinal Youth at Risk Study. A total of 12 individuals converted to psychosis, 15 remitted from UHR status, and 14 maintained UHR status. The responses from the converted group were obtained within 12 months of psychosis onset, while the responses from the remitted and maintained groups were collected at baseline. Linguistic features analyzed included words per minute, articulation rate, dysfluency, and sequential coherence. Acoustic features comprised the mean and SD of fundamental frequency, the mean and SD of intensity, and HF500. Feature differential analysis was conducted via multivariate linear regression. Linear support vector machines were trained as outcome prediction models. Nested cross-validation was used to estimate the generalizability error. The models were principally evaluated on balanced accuracy (BA).

**Results:**

The converted group exhibited lower words per minute (adjusted *P*=.02) and higher dysfluency (adjusted *P*=.004) compared to the remitted group. No significant differences were found in articulation rate, sequential coherence, or acoustic measures across the outcome groups. Two models outperformed random guess, namely the models using linguistic variables (BA 0.741, 95% CI 0.521-0.882) and linguistic and acoustic variables (BA 0.851, 95% CI 0.508-0.944).

**Conclusions:**

Linguistic features extracted from a short speech task exhibit a measurable difference between the outcome groups. Our findings support the feasibility of using signals extracted from the HiSoC task recordings to predict remission in participants with UHR.

## Introduction

Psychosis is typically characterized by hallucinations without insight, delusions, and formal thought disorder [1]. Individuals who experience psychosis often experience a substantial decrease in their quality of life and might require long-term treatment with antipsychotic medication [2,3].

The accurate identification of individuals at heightened risk of developing psychosis is a key component in early intervention to improve clinical outcomes [4]. Many individuals who develop psychosis often exhibit a prodromal phase during which subthreshold symptoms begin to manifest [5,6]. The identification of individuals in this prodromal phase is the basis of ultra-high risk (UHR) assessments such as the Structured Interview for Prodromal Syndrome and the Comprehensive Assessment of At-Risk Mental States [7,8]. However, these psychometric assessments often have high sensitivity but low specificity; that is, most individuals designated as UHR do not go on to develop psychosis [9]. Thus, there is substantial motivation to develop methods to supplement standard UHR assessments. Recently, an impressive range of prediction models has been developed using a variety of modalities, including biomolecular markers, clinical assessments, and linguistic and acoustic analyses [10-16].

Linguistic and acoustic analyses are particularly promising approaches since speech disturbances constitute some of the hallmarks of neurological disturbances and can be observed in most individuals with schizophrenia [17,18]. Deficits such as poverty of speech, greater dysfluency, reduced coherence, derailment, and tangentiality have been consistently reported over the years and form what is now commonly known as “schizophrenia speech” [19-22]. The presence of such deficits is often correlated with limited functioning [23]. Some of these deficits can often be observed at early stages of disease progression, including individuals with UHR [24,25]. Additionally, UHR individuals displaying greater deficits in verbal fluency and coherence are more likely to transition to psychosis [26-28]. There are also linguistic differences between individuals with early stages of schizophrenia and individuals with established schizophrenia, suggesting that the deficits can vary across the disease progression [29]. These varied speech and linguistic deficits are consistently observed across different languages and cultures, including Japanese-, Chinese-, and Portuguese-speaking individuals with UHR [30-34].

Various studies have attempted to combine natural language processing (NLP) methods and machine learning to predict UHR outcomes. One example [10] used open-ended narrative interviews of approximately 1-hour duration from 34 individuals with UHR, with 5 converting to psychosis, to train models using semantic coherence and speech complexity, achieving 100% accuracy in predicting psychosis onset. A more recent work [12] involved developing a predictive model using speech data from the Caplan “story game” along with linguistic markers, such as reduced semantic coherence, increased variance in coherence, and decreased use of possessive pronouns [35]. The study used 93 participants with UHR recruited from 2 sites and achieved 83% accuracy in predicting psychosis onset. Finally, current literature also reported that individuals with UHR with lower connectedness at baseline are more likely to develop affective disorders [33].

These studies suggest potential for the use of NLP methods and machine learning on speech recordings to predict UHR outcomes. Automatic speech recognition (ASR) technologies have advanced substantially in recent years. While much work remains in terms of ensuring the reliable performance of ASR models in real-world applications and in individuals with dysfluent speech [36,37], the general trends are promising—with some models achieving over 90% accuracy in benchmark tests [38,39]. Recent automated speech analysis pipelines have also found some success in predicting an individual’s depression, anxiety, and suicidal ideation level as assessed by self-reported questionnaires [40-42]. With the eventual development of more accurate ASR models, it is conceivable that psychosis risk screens based on automated voice and speech analysis can be developed in the near future. For such screens, long and open-ended speech tasks such as those used in [10,12] might not be scalable as they usually require a lengthy involvement and trained personnel to administer the task. As such, we believe that it is appropriate to explore the predictive potential of speech data extracted from shorter speech tasks. Such findings will be useful in identifying potential tasks that can be more readily used in future automated screens.

The High-Risk Social Challenge (HiSoC) task is designed to assess social functioning in individuals with UHR [43,44]. In the HiSoC task, participants are tasked with providing a 45-second response to a scenario, such as an audition for a competition or a job interview, with minimal preparation. Participant responses are video-recorded and scored on 16 items by trained assessors on a 5-point Likert scale. We previously demonstrated that the HiSoC task can effectively discriminate between individuals with UHR and healthy controls [43,45]. Several properties of the HiSoC task make it a particularly promising source of prognostic information. First, the HiSoC task can be administered quickly, requiring only 10 seconds of preparation and 45 seconds for execution (approximately 1 min total). Second, it is designed to evaluate social functioning, which has been consistently reported to be a strong predictor of clinical outcome [46-48]. Third, the HiSoC task collects video recordings from which audio recordings can be extracted. The speech contents of the recordings can be transcribed, and the acoustic properties of the speech are analyzed to generate a significant amount of data points for research and potential prognostic purposes. Fourth, the HiSoC task only requires a medium through which the prompt can be transmitted and a device to capture a video of the response; both of which can be done using a smartphone. These properties suggest the HiSoC is a task that is potentially suitable for future screens, and there is potential for the screen to be completely remote and automated.

In this study, we perform an exploratory study on the feasibility of using linguistic and acoustic features extracted from HiSoC task recordings to predict outcomes in UHR. The data used in this study were collected as part of the LYRIKS (Longitudinal Youth at Risk Study) [49], an Asian UHR cohort. We examined 2 prediction outcomes, conversion and remission. While the prediction of conversion is of obvious clinical importance, the ability to accurately predict remission is also clinically important, as it allows for individuals who are likely to remit to be assigned to a lower risk group. More intensive intervention can then be directed toward those who are at a higher risk of conversion and maintaining UHR status. Indeed, individuals who maintain UHR status often still experience reduced functioning and long-term attenuated psychotic symptoms[50].

## Methods

### Participants

Participants were recruited as part of the LYRIKS [49]. The LYRIKS is a longitudinal cohort observation study conducted between 2008 and 2010. A total of 2368 individuals were assessed for eligibility, the Comprehensive Assessment of At-Risk Mental States was performed for 926 individuals, and 667 were accepted into the study. The 667 participants consist of 173 participants with UHR and 494 control participants aged between 14 and 29 years. The participants were monitored over a 2-year period between 2008 and 2010. Of the 173 participants with UHR, 17 converted to psychosis (approximately 10% conversion rate). Participants who converted were removed from the study following the collection of the final data point.

Participants assessed to have converted to psychosis were excluded from the study following final data collection. Participants in the LYRIKS were recruited from a mixture of help-seeking and non–help-seeking individuals. Outreach and recruitment strategies are detailed in [51]. All assessments were performed at the same center (Institute of Mental Health, Singapore). The inclusion criteria for the study include (1) aged between 14 and 29 years and (2) English-speaking. Exclusion criteria include (1) having a past or current history of psychosis or intellectual disability, (2) currently using illicit substances, (3) taking antipsychotics or mood stabilizers, (4) having medical causes associated with their psychosis, and (5) contraindications for magnetic resonance imaging. None of the participants were exposed to antipsychotics, mood stabilizers, or illicit substances including cannabis.

Study participants were selected based on the availability of HiSoC recording data, which were collected at 12-month intervals (mo 0, mo 12, and mo 24). Of the 173 participants with UHR, 50 remitted from UHR status within the first 12 months of the study. Among the 17 participants with UHR who transitioned to psychosis, HiSoC task recordings from within the 12 months prior to conversion were available for 12 participants. All 12 recordings were included to form the Converted outcome group. A total of 32 UHR participants did not convert to psychosis but continued to meet the criteria for UHR throughout the duration of the study. HiSoC task recordings from month 0 are available for 14 of them. All 14 recordings were selected to form the maintained outcome group.

HiSoC task recordings from month 0 were available for 28 participants who remitted, and 15 were randomly selected to form the Remitted outcome group. This undersampling was performed to keep the number of individuals in each outcome group proportionally similar to avoid class imbalance issues during the training of predictive modeling classifiers.

### HiSoC Task

Speech recordings used in this study were recorded as part of the HiSoC [43]. Participants were presented with a scenario where they are taking part in a “most interesting person in Singapore” competition, whereby “The winner will be selected based on a 45-second video about themselves.” The participants were given 10 seconds to prepare a response before video recording commenced. The video-recorded response was assessed by 2 trained raters on 16 items each on a 5-point Likert scale. The 16 items can be grouped into 5 domains: affect, social-interpersonal, behavior, and language [44]. The HiSoC task generates a video recording of the participant performing the task, along with the raters’ scoring. All HiSoC tasks were performed in the same study center and recorded using a Sony Handycam DCR SR47 camcorder.

### Covariates

Various covariates were assessed to ensure that the outcome groups do not significantly differ in terms of symptom severity, anxiety, cognition, depression, and education levels. Symptom severity was measured using the Positive and Negative Syndrome Scale (PANSS), which is a clinical assessment of the severity of positive and negative symptoms in individuals with psychosis and UHR [52]. Anxiety was assessed using the Beck Anxiety Inventory (BAI) score, which is the total score across the 21 items of the BAI [53]. Cognitive performance was measured using the Brief Assessment of Cognition in Schizophrenia (BACS), which is an instrument that specifically assesses the aspects of cognition impaired and correlated with clinical outcomes in individuals with schizophrenia [54]. Aspects assessed by the BACS include verbal memory, disorganized speech, token motor task (TMT), verbal fluency, symbol coding, and the Tower of London. The presence of depressive disorder was assessed by whether the individual had an active diagnosis of a depressive disorder [1]. Education level was assessed by 2 measures, namely whether the participant undertook the Primary School Leaving Examination (PSLE) later than expected and whether they have a low education level relative to age. The PSLE is a mandatory national examination taken by all school children at 12 years of age in Singapore. We defined an individual to have late PSLE if they undertook the PSLE after the age of 13 years. Individuals were indicated as having low education relative to age if they had not attained or were currently undergoing postsecondary education by the age of 18 years.

### Transcription

To maximize transcription accuracy, we used manual transcription by 2 independent transcribers (MYL and JK) trained in conversation analysis and transcription methodologies. The transcribers were blinded to the outcome group of the individuals in the recording. These transcribers were not trained in rating the HiSoC task. All identifiable information was removed from transcripts. Transcriber 1 completed all 41 recordings, while transcriber 2 transcribed 12 randomly selected recordings (4 from each outcome group). Consistency between the 2 transcribers was assessed using the Pearson correlation. VLC media player (VideoLAN) was used to extract audio files from the video recording [55]. Speech was performed using PRAAT (version 6.3.15; Boersma and Weenink) [56]. The transcription key used can be found in Table S1 in Multimedia Appendix 1.

The spectrogram was used to support the identification of silent segments, pitch, and intensity variations. Timestamped annotation and transcripts from PRAAT were exported as textgrid files into Python (Python Software Foundation) for feature extraction.

### Linguistic Variables

The following linguistic variables were extracted from the recordings:

1. Words per minute (WPM): the average number of words spoken by participants within 1 minute. However, since the duration of the HiSoC task is fixed at 45 seconds, our version of WPM is determined by multiplying the total number of words spoken during the task by 0.75.
2. Articulation rate (AR)*:* speed of speech production. It is determined by dividing the total word count by the actual speech duration, excluding pauses [57].
3. Dysfluency: the ratio of short or medium pauses, along with the number of interjections, to the total word count in a text. Short pauses are defined as those lasting less than 0.3 seconds, while medium pauses range between 0.3 and 0.7 seconds. Interjections are identified using spaCy’s Part-of-Speech tagging, made available via the “en_core_web_lg” model [58].
4. Sequential coherence (SC)*:* connectedness and similarity between adjacent words. SC is effective in differentiating individuals with schizophrenia from healthy controls and in performing derailment detection [59,60]. Using Word2Vec embeddings from the spaCy en_core_web_lg model, SC is calculated as the mean Word2Vec similarity between adjacent words across the text [58,60]. A moving average with a window of size 5 was used. SC was computed using Word2Vec rather than distribution methods such as latent semantic analysis (LSA) and Latent Dirichlet Allocation, as distributed methods such as Word2Vec were reported to have better performance and more closely match human ratings [61,62].

All linguistic features and their abbreviations are listed in Table 1.

**Table 1.** Name and abbreviation of linguistic and acoustic features.

Type and variable name		Variable abbreviation
Linguistic		
Words per minute		WPM
Articulation rate		AR
Dysfluency		Dysfluency
Sequential coherence		SC
Acoustic		
F0 mean		F0_m
F0 SD		F0_sd
Intensity mean		Int_m
Intensity SD		Int_sd
HF500		HF500

### Acoustic Variables

Intensity (loudness), fundamental frequency F0 (pitch), and spectral energy were extracted from audio recordings and used to derive the following acoustic variables:

1. Fundamental frequency (F0): the rate at which the vocal fold vibrates during speech. Fundamental frequency conveys key elements about the speaker’s identity (different F0 across vowels), sex (lower in males), and emotion (higher and lower F0 when happy and sad, respectively) [63,64]. The *mean fundamental frequency (F0_m*) and *F0 standard deviation (F0_sd*) were extracted from each recording using PRAAT [56]. A high-pass filter at 140 Hz for female participants and 75 Hz for male participants, along with a low-pass filter of 300 Hz for both sexes, was applied.
2. Intensity: the loudness of the voice measured in decibels. We calculated the *mean intensity (Int_m*) and *intensity standard deviation (int_sd*) of the intensity values obtained from PRAAT [56]. These measures allow us to examine whether the different outcome groups exhibit differences in loudness and variations in loudness. Readings below 10 dB were omitted to reduce the effect of ambient sound on the measures.
3. HF500*:* the relative proportion of high-frequency acoustic energy (>500 Hz) to low-frequency acoustic energy (<500 Hz) in the spectrum. This measure has been reported to be a viable measurement of emotional states in voices [65].

All acoustic features and their abbreviations are listed in Table 1.

### Data Processing and Statistical Analysis

Data processing and statistical analysis were conducted in the Python version 3.10 programming environment. The data were standardized prior to statistical testing and predictive modeling. Statistical significance between the outcome groups across covariates was assessed using ANOVA for continuous variables and the chi-square test for binary variables.

Linear regression models were constructed for each linguistic and acoustic feature. To allow for assessments on whether differences in linguistic and acoustic features are associated with depression diagnosis (DD), sex, cognition (BACS), or anxiety (BAI), these covariates are included in the model along with the outcome group (outcome):


y~DD+sex+BACS+BAI+outcome

To examine pairwise differences between the outcome groups, we performed pairwise *t* tests on the outcome groups. Regression analyses were performed using the statsmodels 0.14.4 Python package. Multiple test correction was performed using the Benjamini-Hochberg procedure [66].

### Outcome Prediction Modeling

Logistic regression and support vector machine (SVM) with a linear kernel are 2 commonly used machine learning models [67]. Mathematically, they are related and tend to perform comparably across most tasks [68]. However, there are some studies suggesting that the SVM performs slightly better in imbalanced datasets [69]. Since our predictive modeling task involves class imbalance, we opted to use SVMs in our study. We used linear SVM with balanced class weights from the *scikit-learn* Python package [70]. Given a dataset with N samples and K classes, the balanced class weight $w_{i}$ for class i is implemented as:


$$w_{i}=\frac{N}{Kn_{i}}$$

where $n_{i}$ is the number of samples in class i.

To perform robust model training and evaluation, we used a nested cross-validation setup. This approach leverages an outer leave-one-out cross-validation loop for performance assessment while relying on an inner stratified 5-fold cross-validation loop for hyperparameter tuning. We selected the best-performing model from the inner loop and passed it to the hold-out test sample in the outer loop. Model output consists of the predicted class label.

We repeated the machine learning training process on 5 combinations of features: HiSoC verbal features only (HiSoC_ve), all HiSoC features (HiSoC_all), linguistic features (linguistic), acoustic features (acoustic), and linguistic and acoustic features (linguistic_acoustic). HiSoC_vs features consist of the items with a strong emphasis on participants’ voice: verbal expression, clear communication, fluency of speech, and social anxiety. HiSoC_all consists of all 15 HiSoC items. linguistic_acoustic consists of all linguistic and acoustic features.

Given there are 3 outcome groups, one-vs-all classification was used to transform the task into a binary classification task. Model performances on 2 tasks were examined: predicting conversion outcome in the next 12 months (converted-vs-all) and predicting remission outcome in the next 12 months (remitted-vs-all). The converted-vs-all task consists of 12 converted individuals as the positive class and 29 nonconversion (15 remitted+14 maintained) individuals as the negative class. The remitted-vs-all task consists of 15 remitted individuals as the positive class and 26 nonremitted (12 converted+14 maintained) individuals as the negative class.

### Model Evaluation

Model performance was assessed using balanced accuracy (BA), defined as:


$$BA=\frac{TPR+TNR}{2}$$

where TPR and TNR are the true positive rate and true negative rate, respectively. 95% CIs for BA were constructed based on 1000 bootstrap resamples. Estimates of generalizability error were obtained from the outer fold of the nested cross-validation. 95% CIs are denoted in brackets in the “Results” section.

Common methods to assess overall model performance when significant data imbalances are present include the BA, the Matthew correlation coefficient (MCC), and the precision-recall curve. The precision-recall curve is not suitable for this study as it requires decision probabilities, and decision probabilities in the SVM in *scikit-learn* are derived via Platt scaling, which is a computationally intensive process that will be further compounded by the bootstrapping procedure [70,71]. We chose BA over MCC as it is often impossible to compare MCC of models trained on different datasets—a process necessary to facilitate future validation [72]. In an imbalanced dataset, classifying all samples to the majority class will give a BA of 0.5, which is equivalent to the expected BA of a random guess in a balanced dataset. We define a model performance to be statistically significant if it outperforms a random guess; that is, the lower bound of 95% CI for BA is >0.5.

### Ethical Considerations

Ethical approval for the LYRIKS was provided by the National Healthcare Group’s Domain Specific Review Board (approval: 2009/00167). After a complete description of the study was provided to the participants, written informed consent was obtained. Participants have the ability to opt out of any assessment or terminate participation at any time. Participants were compensated after each visit. All data used were deidentified prior to any analysis. Secondary analyses such as those performed in this study are fully covered under existing ethical approvals and written informed consent from the participants. All researchers involved were required to sign confidentiality and data protection agreements prior to access to the data.

## Results

### Demographics

Across the outcome groups, no significant differences in age, sex (proportion of female participants), PANSS, education (late PSLE and low education relative to age), and BAI scores were observed. Statistically significant differences in BACS TMT across the outcome groups were observed (*F*_2,22_=5.214, *P*=.02; Table 2). The Tukey test revealed that the remitted group exhibited a significantly higher score for BACS_TMT than the Converted group (Table S2 in Multimedia Appendix 1), suggesting that the converted group has much lower motor speed than the remitted group.

**Table 2.** Participant demographics.

Characteristic	Remitted	Maintained	Converted	ANOVA (*P* value)	Chi-square test (*P* value)
Age (y), mean (SD)	22.1 (2.90)	20.6 (4.16)	20.9 (3.75)	.51	—^a^
Sex, n (%)				—	.68
Female	6 (40)	4 (28.6)	3 (25)		
Male	9 (60)	10 (71.4)	9 (75)		
PANSS^b^, mean (SD)					
PANSS +	9.9 (2.99)	10.6 (2.56)	10.7 (2.87)	.72	—
PANSS –	10.6 (4.29)	12.1 (4.37)	12.6 (4.01)	.44	—
Education attainment, n (%)					
Late PSLE^c^	0 (0)	1 (7.14)	0 (0)	—	.38
Low education level relative to age	1 (6.67)	1 (7.14)	2 (16.7)	—	.64
BACS^d^, mean (SD)					
VM^e^	43.1 (7.18)	45.1 (11.64)	43.2 (9.14)	.82	—
DS^f^	21.0 (4.07)	20.4 (3.89)	18.3 (4.01)	.22	—
TMT^g^	76.3 (8.17)	70.1 (12.09)	62.0 (13.86)	.02	—
VF^h^	47.3 (12.75)	41.8 (10.82)	37.4 (11.17)	.10	—
SC^i^	58.3 (10.48)	58.1 (9.48)	52.6 (16.77)	.42	—
TOL^j^	18.0 (1.69)	18.7 (2.20)	16.9 (3.40)	.19	—
Anxiety and depression					
BAI^k^ score, mean (SD)	17.3 (13.15)	16.9 (11.41)	21.3 (15.13)	.67	—
DD^l^, n (%)	4 (26.7)	3 (21.4)	4 (33.3)	—	.79

a Not applicable.

b PANSS: Positive and Negative Syndrome Scale.

c PSLE: Primary School Leaving Examination.

d BACS: Brief Assessment of Cognition in Schizophrenia.

e VM: verbal memory.

f DS: disorganized speech

g TMT: token motor task.

h VF: verbal fluency.

i SC: symbol coding.

j TOL: tower of London.

k BAI: Beck Anxiety Inventory.

l DD: depression diagnosis.

### Linguistic Measures

WPM, AR, dysfluency, and SC measures were consistent between transcribers (*R*^2^=0.993, 0.993, 0.929, and 0.868 for WPM, AR, dysfluency, and SC, respectively; Figure S1A-D in Multimedia Appendix 1), indicating that the transcription and linguistic measures are consistent across transcribers.

WPM was lower in the maintained group relative to the remitted group (*β*=−0.79, 95% CI −1.52 to 0.06; *P*=.04); however, this difference was no longer significant following FDR correction (adjusted *P*=.05). Similarly, WPM was lower in the converted group compared to the remitted group (*β*=−1.17, 95% CI −2.02 to −0.33; *P*=.008). This result remained significant following FDR correction (adjusted *P*=.02). Since AR was not observed to significantly differ between the outcome groups, this reduction in WPM suggests the converted group spoke at a similar speed as the remitted group but spoke significantly fewer words.

**Figure 1.**
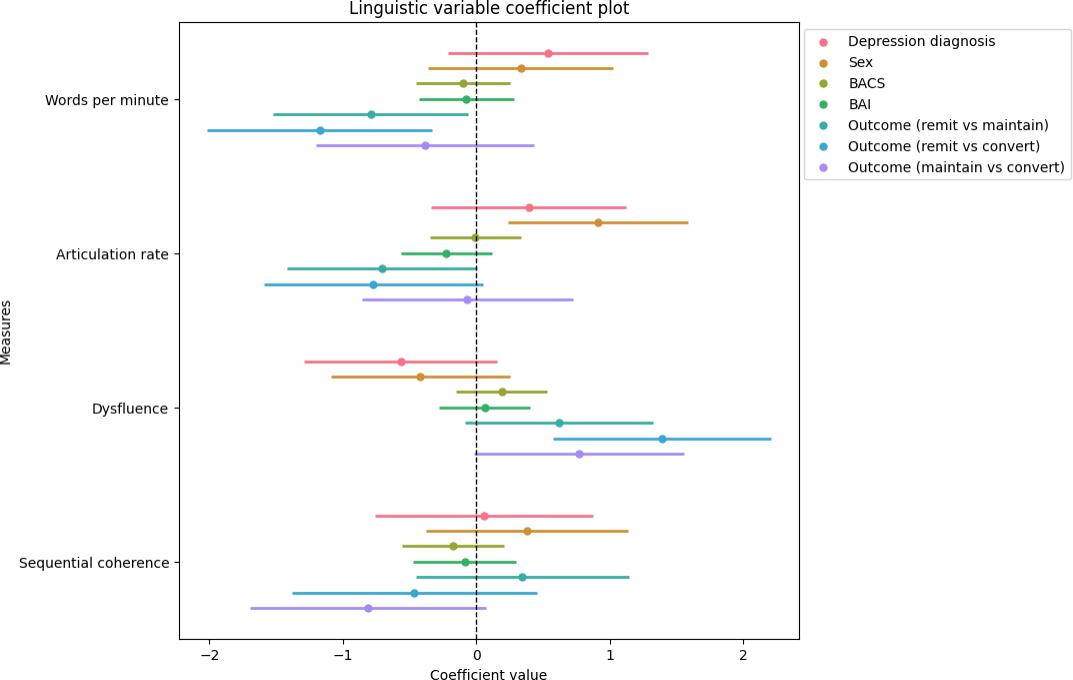
Coefficient plot of each covariate for each linguistic measure (outcome). The coefficients of models fitted to words per minute, articulation rate, dysfluency, and sequential coherence are shown. Each point represents the estimated coefficient for a given predictor-response pair, with horizontal lines indicating the 95% CIs. To facilitate interpretation, we presented coefficients of the outcome group contrasts rather than the coefficients of the outcome group covariates. The covariate is statistically significant if the 95% CI does not intersect 0. BACS: Brief Assessment of Cognition in Schizophrenia; BAI: Beck Anxiety Inventory.

We also observed that the converted group exhibits significantly higher dysfluency relative to the remitted group (*β*=1.39, 95% CI 0.58-2.21; *P*=.001), surviving FDR correction (adjusted *P*=.004; Figure 1), suggesting that the speech of the converted group has significantly more interjections and pauses.

A full table of all coefficients and the associated statistics can be found in Tables S3-S4 in Multimedia Appendix 1.

### Acoustic Measures

**Figure 2.**
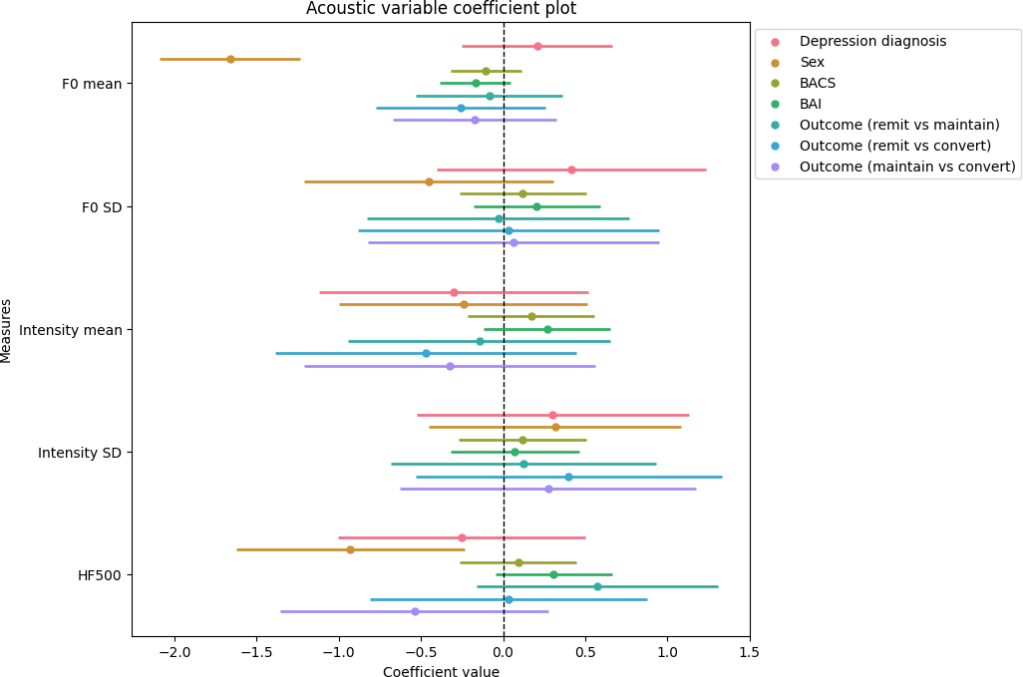
Coefficient plot of each covariate for each acoustic measure (outcome). The coefficients of models fitted to F0_m, F0_sd, Int_m, Int_sd, and HF500 are shown. Coefficient plot of the acoustic measures for acoustic features. Each point represents the estimated coefficient for a given predictor-response pair, with horizontal lines indicating the 95% CIs. To facilitate interpretation, we presented coefficients of the outcome group contrasts rather than the coefficients of the outcome group covariate. The covariate is statistically significant if the 95% CIs do not intersect 0. BACS: Brief Assessment of Cognition in Schizophrenia; BAI: Beck Anxiety Inventory.

We did not observe any significant differences between the outcome groups across all 5 acoustic measures. We observed sex differences in F0 mean and HF500, with male participants exhibiting lower F0 (*β*=−1.66, 95% CI −2.09 to −1.24; *P*<.001) and lower HF500 (*β*=−1.66, 95% CI −2.09 to −1.24; *P*<.001) than female participants (Figure 2). These observations indicate a lower pitch in male participants and a brighter voice quality in female participants. These are expected differences.

A full table of all coefficients and the associated statistics can be found in Tables S5-S6 in Multimedia Appendix 1.

### Outcome Prediction

We examined the performance models trained using HiSoC_all, HiSoC_ve, linguistic, acoustic, and linguistic+acoustic features set across the converted-vs-all and remitted-vs-all tasks.

In the converted-vs-All task, the acoustic model demonstrated the highest BA (BA=0.595, 95% CI 0.282-0.764), followed by the linguistic model (BA=0.570, 95% CI 0.339-0.815) and HiSoC_ve (BA=0.480, 95% CI 0.203-0.774). The HiSoC_all model (BA=0.470, 95% CI 0.310-0.778) and the linguistic+acoustic model (BA=0.529, 95% CI 0.246-0.798) achieved the lowest BAs in this task. However, none of the model performances outperformed a random guess as the lower bounds of the 95% CI of BA were <0.5.

In the remitted-vs-all task, the linguistic+acoustic model achieved the highest balanced accuracy (BA=0.851, 95% CI 0.508-0.944), followed by HiSoC_all (BA=0.760, 95% CI 0.382-0.9), linguistic (BA=0.741, 95% CI 0.521-0.882), and HiSoC_ve (BA=0.645, 95% CI 0.405-0.813). The acoustic model demonstrated the lowest balanced accuracy (BA=0.574, 95% CI 0.325-0.798) in this task. The performances of the linguistic+acoustic model and the linguistic model both outperform a random guess. However, there is substantial overlap between the 95% CI of the 2 models, which means that we cannot determine if there are any meaningful differences in performance between the 2 models.

Regularization parameters and model coefficients are provided in Tables S7-S9 in Multimedia Appendix 1. Specificity and sensitivity of the models are provided in Table S10 in Multimedia Appendix 1.

## Discussion

### Principal Findings

In this study, we explore the outcome prediction potential of linguistic and acoustic features extracted from the HiSoC task. Our findings suggest that linguistic and acoustic features extracted from the HiSoC task contain signals that can potentially differentiate between the outcome groups; most notably, the converted group exhibits lower WPM and higher dysfluency compared to the remitted group. In our prediction task, our linguistic and linguistic+acoustic models achieve good performance (BA=0.741 and 0.851, respectively) and outperformed random guess in the remitted-vs-all task. These findings are promising and support further studies around the use of short speech tasks such as the HiSoC for outcome prediction.

Regression analysis revealed the converted group exhibited lower WPM and higher dysfluency relative to the remitted group. The decrease in WPM in the converted group is indicative of the poverty of content. This reduction is consistent with reduced speech time in individuals with schizophrenia compared to healthy controls [73]. Measures of poverty of speech, both via expert evaluation and NLP methods, have been shown to be predictive of psychosis onset in individuals with UHR [16,74,75]. The increase in dysfluency in the converted group compared to the remitted group is consistent with reports of individuals with UHR who convert to psychosis displaying greater dysfluency compared to those who do not [26,27]. Additionally, greater dysfluency is correlated with increased negative symptom severity, which is in turn correlated with an increased risk of psychosis onset [76,77].

We did not observe any statistically significant differences in SC. This is despite a reduction in semantic coherence being a key predictor of conversion outcome in prior studies [10,12]. We hypothesize two reasons for this difference: (1) this could be due to the length of the HiSoC task being too short to effectively collect sufficient speech output for semantic coherence to be accurately measured. (2) Semantic coherence in this study is measured as SC, which is the average Word2Vec similarity between adjacent words, whereas LSA was used in prior studies [10,12]. The SC method was chosen as Word2Vec had been shown to outperform LSA and is more consistent with human raters than distributional methods such as latent Dirichlet allocation and LSA [61,62]. It is possible that LSA is superior to Word2Vec in this application.

We also did not observe any statistically significant differences between the outcome groups in any of the acoustic measures assessed (F0 mean, F0 SD, intensity mean, intensity SD, and HF500) across the 3 outcome groups. This is despite monotonous speech being a common feature of schizophrenia speech [19]. Meta-analyses of voice patterns in schizophrenia have found that the effect sizes of reduced pitch variability are inconsistent across studies [73], suggesting that, despite monotonous speech being a common feature of schizophrenia speech, reduced pitch variability is not always observed. This could be due to the inherent heterogeneity in the manifestation of speech and language disturbances as well as the nature of the task used to generate the response [78]. The lower F0 mean and HF500 observed in male participants are expected sex differences.

In our prediction tasks, only the linguistic+acoustic model and the linguistic model in the remitted-vs-all task were able to outperform a random guess. This has 2 key implications. First, the primary purpose of this study is to explore the predictive potential of short speech tasks such as the HiSoC. With this result, we found evidence suggesting that linguistic and acoustic features extracted from the HiSoC task can capture speech features that are predictive of remission. Second, none of the models in the converted-vs-all task achieved a performance that is statistically significant, suggesting that the linguistic and acoustic features were able to predict remission but not conversion. Together with the lack of any statistical difference between the converted and maintained groups, it is suggested that the speech patterns of the maintained group do not differ significantly from the converted group within the HiSoC task. If this finding is generalizable, it suggests that the speech patterns of individuals who convert to psychosis and individuals who maintain UHR status are largely similar. Consequently, efforts to predict conversion to psychosis using speech patterns will always be complicated by difficulties in differentiating between individuals who converted and individuals who maintained. A recent study has found that language disturbances are a strong predictor of response to clinical interventions; individuals with UHR with lower levels of language disturbances exhibit greater improvement in both symptom severity and functioning over time [50]. It is possible that speech and language disturbances more accurately reflect individual capacity for improvement rather than eventual clinical outcome. With these considerations, predicting remission from UHR status might be a more feasible direction than predicting conversion to psychosis. The ability to identify individuals likely to remit still has tremendous use as it allows for greater focus to be placed on those not likely to remit, allowing limited resources to be distributed to those who need them the most.

While our findings indicate that signals extracted from the HiSoC task can feasibly be used to predict remission, it must be reiterated that the study is intended to be exploratory and that any findings are exploratory and limited by the small sample size. Even so, signals are still strong enough to be detected. Future validation studies with larger independent datasets are necessary to validate both the findings and model generalizability before clinical or screening implications can reasonably be considered.

This study examines predictive potential involving speech data extracted from the HiSoC task. However, while there are several tasks designed to elicit speech in mental health, there is little consistency in the tasks used. For example, tasks used in recently published automated speech analysis pipelines include reading from selected passages [40], semistructured speech tasks such as “Describe how you are feeling at the moment and how your nights’ sleep have been lately” [42], and talking to research nurses [41]. A comparative study using a variety of speech tasks should be performed to examine whether the outcome group differences are consistent across different tasks, and if there is an optimal task for outcome prediction.

While ASR promises scalability that can potentially unlock fast and efficient automated speech-based risk screens, current ASR models tend to exhibit higher error rates in dysfluent speech [36,37]. This might be particularly problematic in psychosis risk screens, where dysfluency is a feature of schizophrenia speech. ASR technologies will likely need to reach a sufficiently reliable and consistent accuracy before an automated psychosis risk screen can achieve sufficient reliability.

### Strengths

To our knowledge, this is the first study diving into the predictive potential of linguistic and acoustic features extracted from audio recordings of the HiSoC task. The recordings used in this study are significantly shorter and more scalable than those in comparable studies [10,12]. While significant validation work remains, we showed that features from the HiSoC task contain statistically significant differences between the outcome groups and that extracted linguistic and acoustic features can be used to predict remission.

Our findings suggest that further exploration into the predictive use of short speech tasks such as the HiSoC in speech analysis is warranted. We expect that this study will be one of the first of many that explore or validate the predictive use of various short speech tasks to facilitate future speech−based automated risk screening tools.

### Limitations

First, although convenient, the short duration of the HiSoC task can potentially lead to data that are less representative of the individual’s speech pattern. As described previously, this might explain the lack of differences in SC between the outcome groups. Additional studies comparing longer open-ended speech tasks and shorter tasks like the HiSoC will be necessary to assess whether shorter tasks sufficiently capture the individual’s speech patterns. Second, our sample sizes are limited by the undersampling performed to keep the number of individuals in each outcome group relatively balanced to minimize class imbalance issues. This meant that our sample size would be limited by the number of participants who converted to psychosis even when more data from individuals who remitted or maintained were available. A small sample size leads to lower statistical power of our regression analysis, which means that there might be differences between the outcome groups that were not detected due to the low statistical power of the test. The large 95% CIs for balanced accuracy in our models are likely a consequence of the small sample size, as the performance of the model can fluctuate significantly depending on the bootstrap resample. A small sample size can also lead to the creation of biased models that do not generalize well. However, the purpose of this study is to explore the potential of developing outcome prediction models using features extracted from the HiSoC task audio recordings and not to develop a definitive model. Third, we lack an independent validation dataset. This limits our ability to accurately estimate generalizability error. It is possible that any class separation within the feature space used in this study is unique to this dataset. A follow-up study using the same feature sets and methods on a comparable dataset is necessary to validate both the regression analysis findings and the model performances.

## Supplementary material

10.2196/75960 Multimedia Appendix 1 Transcription keys, interrater reliability, regression summaries, and model weights.

## Abbreviations

AR: articulation rate
ASR: automatic speech recognition
BA: balanced accuracy
BACS: Brief Assessment of Cognition in Schizophrenia
BAI: Beck Anxiety Inventory
DD: depression diagnosis
DF: dysfluency
GAI: generative artificial intelligence
HiSoC: High-Risk Social Challenge
LSA: latent semantic analysis
LYRIKS: Longitudinal Youth at Risk Study
MCC: Matthew correlation coefficient
NLP: natural language processing
PANSS: Positive and Negative Syndrome Scale
PSLE: Primary School Leaving Examination
SC: sequential coherence
SVM: support vector machine
TMT: token motor task
UHR: ultra-high risk
WPM: words per minute
