# Supplementary material for: Predicting Ultra-High Risk Outcomes Using Linguistic and Acoustic Measures From High-Risk Social Challenge Recordings: mHealth Longitudinal Cohort Exploratory Study
Source: JMIR Form Res. 2025 Dec 30;9:e75960. doi: 10.2196/75960 (PMC12753029; doi:10.2196/75960)
Supplement: Multimedia Appendix 1 [file formative-v9-e75960-s001.docx]

**Predicting UHR Outcomes Using Linguistic and Acoustic Measures from HiSoC Recordings: A mHealth Longitudinal Cohort Exploratory Study**

**Supplementary materials**

Samuel Tan^1,^^, Lieu May Yen^1^, Jun Kai^2^, Zixu Yang^3^, K.K. Luke^2^, May Oo Lwin^1,4^, Jimmy Lee^1,3*^, Wilson Wen Bin Goh ^1,5,6,7,8*^

1. Lee Kong Chian School of Medicine, Nanyang Technological University, Singapore
2. School of Humanities, Nanyang Technological University, Singapore
3. Institute of Mental Health, Singapore
4. Wee Kim Wee School of Communication and Information, Nanyang Technological University, Singapore
5. School of Biological Sciences, Nanyang Technological University, Singapore
6. Centre for Biomedical Informatics, Nanyang Technological University, Singapore
7. Centre of AI in Medicine, Nanyang Technological University, Singapore
8. Division of Neurology, Department of Brain Sciences, Faculty of Medicine, Imperial College London

^ First Author

* Corresponding Author(s): Jimmy Lee, [jimmy_lee@imh.com.sg](mailto:jimmy_lee@imh.com.sg); Wilson Wen Bin Goh, [wilsongoh@ntu.edu.sg](mailto:wilsongoh@ntu.edu.sg)

**Supplementary Table 1:** transciption keys

| Category | Symbol |
| --- | --- |
| Speaker label | Participant (P)  Interviewer (I) |
| Word cut off | Dash (-) |
| Redacted information  *Name, home address, phone number, D.O.B, etc.* | * (to replace each syllable)  e.g.,  John 🡪 *  Teddy 🡪 ** |
| Words (notable deviation from standard pronunciation, as well as any Singlish utterances) | Sweet <[swiːt]> |
| Uncertain hearing | X (for each indecipherable syllable)  <X>  </ X> (to surround word or syllable that represent the transcriber’s ‘best guess’) |
| Final tone | Full stop (.) |
| Continuing tone | Comma (,) |
| Rising tone of uncertainty | Question mark (?) |
| Transcriber’s comments | Square brackets [ ] |
| Stress | Word surrounded by dashes  e.g., I -want- to talk |
| Utterance | <S> for any vocalization |
| Silence | <Z> |
| Pauses | Micro pause (less than 0.3s): .. (two dots)  Medium pause (between 0.3 to 0.7 s): ... (three dots, also marked as (.) in the segment tier)  Long pause (more than 0.7s) : ..... (four dots, also marked as <Z> in the segment tier) |
| Lengthening | Equal sign following lengthened segment (yea=) |
| Vocal noise | Inbreath : (H)  Outbreath: (Hx)  Laughter: @ (for each ‘syllable’ of laughter) |
| Vocal quality | Loud : <LOUD>   </LOUD>  Soft: <SOFT> </SOFT>  High pitch: <HI> </HI>  Low pitch: <LO> </LO>  Fast: <FAST> </FAST>  Slow: <SLOW> </SLOW>  Syllable rise in pitch: _↑  Syllable fall in pitch: _↓ |

**Supplementary Table 2:** BACS_TMT Tukey’s HSD

| Comparison | Statistic | p-value | Lower CI | Upper CI |
| --- | --- | --- | --- | --- |
| RMT - CVT | 14.267 | 0.007 | 3.49 | 25.043 |
| RMT – MNT | 6.124 | 0.329 | -4.217 | 16.464 |
| CVT - MNT | -8.143 | 0.179 | -19.089 | 2.804 |


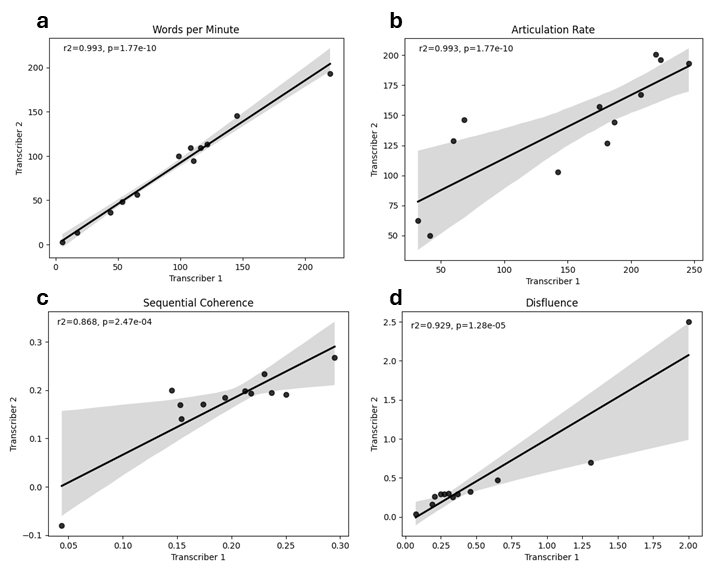


**Supplementary Figure 1.** Regression plot of linguistic variables **(a)** WPM, **(b)** AR, **(c)** SC, and **(d)** DF. Across all four variables, the regression lines indicate a strong positive relationship p < 0.001. Indicating high consistency across transcribers. Additionally, the correlation coefficients are substantial (>0.86 in SC and > 0.9 in WPM, AR, and DF)

**Supplementary Table 3a:** model summary (WPM)

|  | coef | std err | T | P>\|t\| | **95% CI Lower** | **95% CI Upper** |
| --- | --- | --- | --- | --- | --- | --- |
| Intercept | 0.2263 | 0.332 | 0.681 | 0.501 | -0.45 | 0.902 |
| C(dep_c)[T.1] | 0.5377 | 0.369 | 1.457 | 0.154 | -0.213 | 1.288 |
| C(gend)[T.2] | 0.3338 | 0.342 | 0.977 | 0.335 | -0.361 | 1.029 |
| C(group_num)[T.1] | -0.7905 | 0.36 | -2.197 | 0.035 | -1.523 | -0.059 |
| C(group_num)[T.2] | -1.1739 | 0.415 | -2.831 | 0.008 | -2.018 | -0.33 |
| bacs | -0.0978 | 0.174 | -0.563 | 0.577 | -0.451 | 0.255 |
| bai | -0.0735 | 0.174 | -0.422 | 0.676 | -0.428 | 0.281 |

**Supplementary Table 3b:** model summary (DF)

|  | **coef** | **std err** | **T** | **P>\|t\|** | **95% CI Lower** | **95% CI Upper** |
| --- | --- | --- | --- | --- | --- | --- |
| Intercept | -0.1617 | 0.321 | -0.504 | 0.617 | -0.814 | 0.491 |
| C(dep_c)[T.1] | -0.5652 | 0.356 | -1.587 | 0.122 | -1.29 | 0.159 |
| C(gend)[T.2] | -0.4207 | 0.33 | -1.276 | 0.211 | -1.091 | 0.25 |
| C(group_num)[T.1] | 0.6224 | 0.347 | 1.792 | 0.082 | -0.084 | 1.329 |
| C(group_num)[T.2] | 1.3939 | 0.4 | 3.482 | 0.001 | 0.58 | 2.208 |
| bacs | 0.1923 | 0.168 | 1.148 | 0.259 | -0.149 | 0.533 |
| bai | 0.0633 | 0.168 | 0.377 | 0.709 | -0.279 | 0.405 |

**Supplementary Table 3c:** model summary (AR)

|  | **coef** | **std err** | **t** | **P>\|t\|** | **95% CI Lower** | **95% CI Upper** |
| --- | --- | --- | --- | --- | --- | --- |
| Intercept | -0.2673 | 0.323 | -0.828 | 0.414 | -0.924 | 0.389 |
| C(dep_c)[T.1] | 0.3924 | 0.358 | 1.095 | 0.282 | -0.337 | 1.122 |
| C(gend)[T.2] | 0.916 | 0.332 | 2.761 | 0.009 | 0.241 | 1.591 |
| C(group_num)[T.1] | -0.7053 | 0.35 | -2.018 | 0.052 | -1.416 | 0.006 |
| C(group_num)[T.2] | -0.7712 | 0.403 | -1.914 | 0.064 | -1.591 | 0.048 |
| bacs | -0.0063 | 0.169 | -0.037 | 0.97 | -0.349 | 0.337 |
| bai | -0.223 | 0.169 | -1.318 | 0.197 | -0.567 | 0.121 |

**Supplementary Table 3d:** model summary (SC)

|  | **coef** | **std err** | **t** | **P>\|t\|** | **95% CI Lower** | **95% CI Upper** |
| --- | --- | --- | --- | --- | --- | --- |
| Intercept | -0.2648 | 0.362 | -0.732 | 0.469 | -1.001 | 0.471 |
| C(dep_c)[T.1] | 0.057 | 0.402 | 0.142 | 0.888 | -0.76 | 0.874 |
| C(gend)[T.2] | 0.3797 | 0.372 | 1.021 | 0.315 | -0.377 | 1.136 |
| C(group_num)[T.1] | 0.3455 | 0.392 | 0.882 | 0.384 | -0.452 | 1.143 |
| C(group_num)[T.2] | -0.4657 | 0.452 | -1.031 | 0.31 | -1.384 | 0.453 |
| bacs | -0.1742 | 0.189 | -0.922 | 0.363 | -0.559 | 0.21 |
| bai | -0.0876 | 0.19 | -0.462 | 0.647 | -0.473 | 0.298 |

**Supplementary Table 4a:** Outcome group contrast statistics (WPM)

| **Contrast** | **Coefficient** | **Std. Error** | **t‑value** | **P‑value** | **95% CI Lower** | **95% CI Upper** | **p‑value (FDR‑BH)** |
| --- | --- | --- | --- | --- | --- | --- | --- |
| 1 − 0 | −0.790529 | 0.359791 | −2.197191 | 0.035135 | −1.522529 | −0.058529 | 0.052702 |
| 2 − 0 | −1.173870 | 0.414716 | −2.830537 | 0.007852 | −2.017617 | −0.330123 | 0.023557 |
| 2 − 1 | −0.383341 | 0.400579 | −0.956968 | 0.345542 | −1.198324 | 0.431642 | 0.345542 |

**Supplementary Table 4b:** Outcome group contrast statistics (DF)

| **Contrast** | **Coefficient** | **Std. Error** | **t‑value** | **P‑value** | **95% CI Lower** | **95% CI Upper** | **p‑value (FDR‑BH)** |
| --- | --- | --- | --- | --- | --- | --- | --- |
| 1 − 0 | 0.622367 | 0.347258 | 1.792231 | 0.082269 | −0.084135 | 1.328868 | 0.082269 |
| 2 − 0 | 1.393941 | 0.40027 | 3.482499 | 0.001422 | 0.579585 | 2.208297 | 0.004265 |
| 2 − 1 | 0.771575 | 0.386625 | 1.995666 | 0.054279 | −0.015020 | 1.558169 | 0.081418 |

**Supplementary Table 4c:** Outcome group contrast statistics (AR)

| **Contrast** | **Coefficient** | **Std. Error** | **t‑value** | **P‑value** | **95% CI Lower** | **95% CI Upper** | **p‑value (FDR‑BH)** |
| --- | --- | --- | --- | --- | --- | --- | --- |
| 1 − 0 | −0.705301 | 0.349509 | −2.017977 | 0.051784 | −1.416382 | 0.00578 | 0.096425 |
| 2 − 0 | −0.771213 | 0.402865 | −1.914322 | 0.064283 | −1.590848 | 0.048422 | 0.096425 |
| 2 − 1 | −0.065912 | 0.389131 | −0.169383 | 0.86653 | −0.857605 | 0.725781 | 0.86653 |

**Supplementary Table 4d:** Outcome group contrast statistics (SC)

| **Contrast** | **Coefficient** | **Std. Error** | **t‑value** | **P‑value** | **95% CI Lower** | **95% CI Upper** | **p‑value (FDR‑BH)** |
| --- | --- | --- | --- | --- | --- | --- | --- |
| 1 − 0 | 0.345454 | 0.391772 | 0.881773 | 0.384278 | −0.451612 | 1.14252 | 0.384278 |
| 2 − 0 | −0.465742 | 0.45158 | −1.031362 | 0.309871 | −1.384489 | 0.453004 | 0.384278 |
| 2 − 1 | −0.811196 | 0.436186 | −1.859751 | 0.071855 | −1.698623 | 0.07623 | 0.215565 |

**Supplementary Table 5a:** model summary (F0_m)

|  | **coef** | **std err** | **t** | **P>\|t\|** | **95% CI Lower** | **95% CI Upper** |
| --- | --- | --- | --- | --- | --- | --- |
| Intercept | 1.1659 | 0.203 | 5.731 | 0 | 0.752 | 1.58 |
| C(dep_c)[T.1] | 0.2064 | 0.226 | 0.914 | 0.367 | -0.253 | 0.666 |
| C(gend)[T.2] | -1.6615 | 0.209 | -7.946 | 0 | -2.087 | -1.236 |
| C(group_num)[T.1] | -0.0851 | 0.22 | -0.386 | 0.702 | -0.533 | 0.363 |
| C(group_num)[T.2] | -0.2595 | 0.254 | -1.022 | 0.314 | -0.776 | 0.257 |
| bacs | -0.104 | 0.106 | -0.978 | 0.335 | -0.32 | 0.112 |
| bai | -0.1696 | 0.107 | -1.591 | 0.121 | -0.387 | 0.047 |

**Supplementary Table 5b:** model summary (F0_sd)

|  | **coef** | **std err** | **t** | **P>\|t\|** | **95% CI Lower** | **95% CI Upper** |
| --- | --- | --- | --- | --- | --- | --- |
| Intercept | 0.1919 | 0.362 | 0.531 | 0.599 | -0.544 | 0.928 |
| C(dep_c)[T.1] | 0.415 | 0.402 | 1.033 | 0.309 | -0.402 | 1.232 |
| C(gend)[T.2] | -0.4512 | 0.372 | -1.214 | 0.233 | -1.208 | 0.305 |
| C(group_num)[T.1] | -0.0304 | 0.392 | -0.078 | 0.939 | -0.827 | 0.766 |
| C(group_num)[T.2] | 0.0334 | 0.451 | 0.074 | 0.942 | -0.885 | 0.952 |
| bacs | 0.119 | 0.189 | 0.63 | 0.533 | -0.265 | 0.503 |
| bai | 0.2056 | 0.19 | 1.085 | 0.286 | -0.18 | 0.591 |

**Supplementary Table 5c:** model summary (int_m)

|  | **coef** | **std err** | **t** | **P>\|t\|** | **95% CI Lower** | **95% CI Upper** |
| --- | --- | --- | --- | --- | --- | --- |
| Intercept | 0.4261 | 0.361 | 1.179 | 0.247 | -0.309 | 1.161 |
| C(dep_c)[T.1] | -0.2998 | 0.401 | -0.747 | 0.46 | -1.116 | 0.517 |
| C(gend)[T.2] | -0.2425 | 0.372 | -0.653 | 0.518 | -0.998 | 0.513 |
| C(group_num)[T.1] | -0.1446 | 0.391 | -0.369 | 0.714 | -0.941 | 0.652 |
| C(group_num)[T.2] | -0.4702 | 0.451 | -1.042 | 0.305 | -1.388 | 0.448 |
| bacs | 0.1712 | 0.189 | 0.907 | 0.371 | -0.213 | 0.555 |
| bai | 0.2678 | 0.189 | 1.413 | 0.167 | -0.118 | 0.653 |

**Supplementary Table 5d:** model summary (int_sd)

|  | **coef** | **std err** | **t** | **P>\|t\|** | **95% CI Lower** | **95% CI Upper** |
| --- | --- | --- | --- | --- | --- | --- |
| Intercept | -0.4515 | 0.367 | -1.231 | 0.227 | -1.198 | 0.295 |
| C(dep_c)[T.1] | 0.3016 | 0.407 | 0.74 | 0.464 | -0.527 | 1.13 |
| C(gend)[T.2] | 0.318 | 0.377 | 0.843 | 0.405 | -0.449 | 1.085 |
| C(group_num)[T.1] | 0.1254 | 0.397 | 0.316 | 0.754 | -0.683 | 0.934 |
| C(group_num)[T.2] | 0.3999 | 0.458 | 0.873 | 0.389 | -0.532 | 1.331 |
| bacs | 0.1188 | 0.192 | 0.62 | 0.539 | -0.271 | 0.509 |
| bai | 0.0709 | 0.192 | 0.368 | 0.715 | -0.32 | 0.462 |

**Supplementary Table 5e:** model summary (hf500)

|  | **coef** | **std err** | **t** | **P>\|t\|** | **95% CI Lower** | **95% CI Upper** |
| --- | --- | --- | --- | --- | --- | --- |
| Intercept | 0.4871 | 0.333 | 1.465 | 0.152 | -0.189 | 1.164 |
| C(dep_c)[T.1] | -0.2537 | 0.369 | -0.687 | 0.497 | -1.005 | 0.497 |
| C(gend)[T.2] | -0.9288 | 0.342 | -2.717 | 0.01 | -1.624 | -0.233 |
| C(group_num)[T.1] | 0.5728 | 0.36 | 1.591 | 0.121 | -0.16 | 1.305 |
| C(group_num)[T.2] | 0.0331 | 0.415 | 0.08 | 0.937 | -0.811 | 0.878 |
| bacs | 0.0908 | 0.174 | 0.523 | 0.604 | -0.263 | 0.444 |
| bai | 0.3061 | 0.174 | 1.756 | 0.088 | -0.048 | 0.661 |

**Supplementary Table 6a:** Outcome group contrast statistics (F0_m)

| **Contrast** | **Coefficient** | **Std. Error** | **t‑value** | **P‑value** | **95% CI Lower** | **95% CI Upper** | **p‑value (FDR‑BH)** |
| --- | --- | --- | --- | --- | --- | --- | --- |
| 1 − 0 | −0.085106 | 0.220285 | −0.386345 | 0.701721 | −0.533280 | 0.363068 | 0.701721 |
| 2 − 0 | −0.259489 | 0.253914 | −1.021957 | 0.314235 | −0.776081 | 0.257103 | 0.701721 |
| 2 − 1 | −0.174383 | 0.245258 | −0.711019 | 0.482068 | −0.673364 | 0.324598 | 0.701721 |

**Supplementary Table 6b:** Outcome group contrast statistics (F0_sd)

| **Contrast** | **Coefficient** | **Std. Error** | **t‑value** | **P‑value** | **95% CI Lower** | **95% CI Upper** | **p‑value (FDR‑BH)** |
| --- | --- | --- | --- | --- | --- | --- | --- |
| 1 − 0 | −0.030416 | 0.391629 | −0.077665 | 0.938564 | −0.827190 | 0.766359 | 0.94153 |
| 2 − 0 | 0.033363 | 0.451415 | 0.073907 | 0.94153 | −0.885047 | 0.951773 | 0.94153 |
| 2 − 1 | 0.063779 | 0.436026 | 0.146273 | 0.884596 | −0.823323 | 0.95088 | 0.94153 |

**Supplementary Table 6c:** Outcome group contrast statistics (int_m)

| **Contrast** | **Coefficient** | **Std. Error** | **t‑value** | **P‑value** | **95% CI Lower** | **95% CI Upper** | **p‑value (FDR‑BH)** |
| --- | --- | --- | --- | --- | --- | --- | --- |
| 1 − 0 | −0.144602 | 0.391389 | −0.369459 | 0.714146 | −0.940890 | 0.651685 | 0.714146 |
| 2 − 0 | −0.470228 | 0.451139 | −1.042312 | 0.304843 | −1.388076 | 0.447621 | 0.690298 |
| 2 − 1 | −0.325625 | 0.435759 | −0.747259 | 0.460199 | −1.212185 | 0.560934 | 0.690298 |

**Supplementary Table 6d:** Outcome group contrast statistics (int_sd)

| **Contrast** | **Coefficient** | **Std. Error** | **t‑value** | **P‑value** | **95% CI Lower** | **95% CI Upper** | **p‑value (FDR‑BH)** |
| --- | --- | --- | --- | --- | --- | --- | --- |
| 1 − 0 | 0.125385 | 0.397263 | 0.315622 | 0.754277 | −0.682852 | 0.933622 | 0.754277 |
| 2 − 0 | 0.399874 | 0.457909 | 0.873262 | 0.388831 | −0.531748 | 1.331496 | 0.754277 |
| 2 − 1 | 0.274489 | 0.442298 | 0.620597 | 0.539127 | −0.625374 | 1.174352 | 0.754277 |

**Supplementary Table 6e:** Outcome group contrast statistics (hf500)

| **Contrast** | **Coefficient** | **Std. Error** | **t‑value** | **P‑value** | **95% CI Lower** | **95% CI Upper** | **p‑value (FDR‑BH)** |
| --- | --- | --- | --- | --- | --- | --- | --- |
| 1 − 0 | 0.572794 | 0.360088 | 1.590706 | 0.121211 | −0.159810 | 1.305397 | 0.281108 |
| 2 − 0 | 0.03308 | 0.415059 | 0.079699 | 0.936958 | −0.811363 | 0.877523 | 0.936958 |
| 2 − 1 | −0.539714 | 0.400909 | −1.346224 | 0.187405 | −1.355370 | 0.275942 | 0.281108 |

**Supplementary Table 7:** Regularization parameters

| **Model** | **C Value** |
| --- | --- |
| cvt_12m_hisoc_ve | 0.021544 |
| remit_12m_hisoc_ve | 0.1 |
| cvt_12m_hisoc_full | 0.004642 |
| remit_12m_hisoc_full | 2.154435 |
| cvt_12m_linguistic | 0.1 |
| remit_12m_linguistic | 2.154435 |
| cvt_12m_acoustic | 46.41589 |
| remit_12m_acoustic | 2.154435 |
| cvt_12m_linguistic_acoustic | 0.464159 |
| remit_12m_linguistic_acoustic | 0.1 |

**Supplementary Table 8:** CVT-vs-ALL model coefficients

| Model | hisoc_ve | hisoc_full | linguistic | acoustic | linguistic_acoustic |
| --- | --- | --- | --- | --- | --- |
| hisoc_01 | - | -0.03279 | - | - | - |
| hisoc_02 | - | -0.03367 | - | - | - |
| hisoc_03 | - | -0.0666 | - | - | - |
| hisoc_04 | -0.19322 | -0.04819 | - | - | - |
| hisoc_05 | - | -0.05709 | - | - | - |
| hisoc_06 | - | -0.0334 | - | - | - |
| hisoc_07 | - | -0.07903 | - | - | - |
| hisoc_08 | - | -0.04337 | - | - | - |
| hisoc_09 | - | -0.04103 | - | - | - |
| hisoc_10 | - | -0.03082 | - | - | - |
| hisoc_11 | -0.09963 | -0.03308 | - | - | - |
| hisoc_12 | -0.18044 | -0.05002 | - | - | - |
| hisoc_13 | - | -0.06016 | - | - | - |
| hisoc_14 | -0.17536 | -0.04495 | - | - | - |
| hisoc_15 | - | -0.0358 | - | - | - |
| words per minute | - | - | -0.35379 | - | -0.28295 |
| proportion of pauses and interjections | - | - | 0.35958 | - | 0.2491 |
| word speed | - | - | -0.08991 | - | -0.42818 |
| coherence | - | - | 0.12807 | - | 0.09689 |
| f0_mean | - | - | - | -0.36848 | -0.44888 |
| f0_sd | - | - | - | 0.25115 | 0.18634 |
| intensity_mean | - | - | - | -0.95867 | -0.41398 |
| intensity_sd | - | - | - | 0.88221 | 0.87654 |
| hf500 | - | - | - | -0.22682 | -0.16664 |

**Supplementary Table 9**: RMT-vs-ALL model coefficients

| Model | hisoc_ve | hisoc_full | linguistic | acoustic | linguistic_acoustic |
| --- | --- | --- | --- | --- | --- |
| hisoc_01 | - | 1.20745 | - | - | - |
| hisoc_02 | - | -1.12715 | - | - | - |
| hisoc_03 | - | -0.42845 | - | - | - |
| hisoc_04 | 0.36576 | 0.58373 | - | - | - |
| hisoc_05 | - | 1.42691 | - | - | - |
| hisoc_06 | - | -0.30799 | - | - | - |
| hisoc_07 | - | 0.33533 | - | - | - |
| hisoc_08 | - | 0.54248 | - | - | - |
| hisoc_09 | - | 0.30907 | - | - | - |
| hisoc_10 | - | -0.88074 | - | - | - |
| hisoc_11 | -0.08086 | -0.65722 | - | - | - |
| hisoc_12 | 0.35476 | -0.14319 | - | - | - |
| hisoc_13 | - | 0.32328 | - | - | - |
| hisoc_14 | 0.26394 | 0.04011 | - | - | - |
| hisoc_15 | - | 1.49137 | - | - | - |
| words per minute | - | - | -0.36715 | - | 0.39005 |
| proportion of pauses and interjections | - | - | -2.26688 | - | -0.57007 |
| word speed | - | - | 0.31476 | - | 0.18115 |
| coherence | - | - | -1.04137 | - | -0.43555 |
| f0_mean | - | - | - | 0.47253 | 0.37023 |
| f0_sd | - | - | - | 0.12014 | -0.23038 |
| intensity_mean | - | - | - | 0.88597 | 0.26517 |
| intensity_sd | - | - | - | -0.35721 | -0.24199 |
| hf500 | - | - | - | -0.73673 | -0.37616 |

**Supplementary Table 10**: Specificity and Sensitivity of models

| **Model** | **Balanced accuracy** | **Sensitivity** | **Specificity** |
| --- | --- | --- | --- |
| remit_12m_hisoc_ve | 0.644872 | 0.866667 | 0.423077 |
| remit_12m_hisoc_full | 0.760256 | 0.866667 | 0.653846 |
| remit_12m_linguistic | 0.741026 | 0.866667 | 0.615385 |
| remit_12m_acoustic | 0.574359 | 0.533333 | 0.615385 |
| remit_12m_linguistic_acoustic | 0.851282 | 0.933333 | 0.769231 |
| cvt_12m_hisoc_ve | 0.479885 | 0.166667 | 0.793103 |
| cvt_12m_hisoc_full | 0.469828 | 0.25 | 0.689655 |
| cvt_12m_linguistic | 0.570402 | 0.416667 | 0.724138 |
| cvt_12m_acoustic | 0.594828 | 0.5 | 0.689655 |
